# Cerebral lipiodol embolism after transcatheter arterial chemoembolization for hepatocellular carcinoma: a case report and review of the literature

**DOI:** 10.3389/fonc.2026.1797796

**Published:** 2026-04-28

**Authors:** Zhifeng Xu, Huawen Xia

**Affiliations:** Department of Interventional Vascular Surgery, Handan First Hospital, Handan, China

**Keywords:** cerebral lipiodol embolism, hepatocellular carcinoma, inferior phrenic artery, transcatheter arterial chemoembolization, vascular lake

## Abstract

Cerebral lipiodol embolism (CLE) is a rare and serious complication of transcatheter arterial chemoembolization (TACE). A 48-year-old male patient underwent his first TACE for a huge hepatocellular carcinoma (HCC). Angiography revealed a vascular lake. The day after the procedure, the patient developed drowsiness accompanied by generalized weakness, and an inability to stand and walk. Cerebral computed tomography (CT) demonstrated multiple hyperdense lesions in the bilateral cerebral and cerebellar hemispheres, with no infarct foci identified—findings consistent with CLE. After treatment with intravenous fat emulsion and other supportive measures, the patient’s neurological condition gradually improved and his walking ability returned to normal. Follow-up cranial CT revealed a significant reduction in parenchymal hyperdensities compared with prior imaging. Although CLE is a rare complication, clinicians should remain vigilant for its occurrence during TACE in patients with high-risk factors.

## Introduction

HCC is the most common histological type of primary liver cancer (PLC), accounting for 75%-85% (1, 2). In China, 84.4% of HCC is attributed to chronic hepatitis B virus (HBV) infection, which stands as the primary cause of HCC (3). In contrast, hepatitis C virus (HCV) infection is the predominant cause in European and American countries (4). TACE has emerged as the preferred approach for transarterial interventional treatment of intermediate and advanced PLC, owing to its advantages such as minimal invasiveness, rapid recovery, and favorable therapeutic outcomes. However, with the widespread application of lipiodol in interventional therapies, reports of lipiodol ectopic embolism have gradually increased, such as pulmonary lipiodol embolism (PLE) and CLE (5). The incidence of PLE is 8.71% (6), whereas that of CLE is less than 0.01% (7).

During angiography in the TACE procedure for this case, it was observed that the HCC was supplied by both the right hepatic artery (RHA) and the right inferior phrenic artery (RIPA). Following injection of the lipiodol mixture, vascular lakes became evident. However, the presence of these vascular lakes was not identified intraoperatively, and embolization was continued. Postoperatively, the patient developed CLE. This report presents the clinical and imaging manifestations of this condition. To further investigate the potential pathogenesis of CLE, we conducted a systematic review and analysis of 49 documented cases identified from relevant literature.

## Clinical data

A 48-year-old male patient presented to our hospital with a chief complaint of intermittent sharp pain in the right upper abdomen that had persisted for 3 months. He had a 30-year history of smoking (10 cigarettes per day) and alcohol use (200 mL of liquor per day), with no cessation prior to admission. He also had a documented history of chronic HBV infection lasting over 10 years, during which he had not received oral antiviral therapy. Laboratory findings included: hemoglobin, 85 g/L; albumin, 32.9 g/L; creatinine, 60 μmol/L. Coagulation profile: prothrombin time, 14.4 seconds; prothrombin activity, 79%; antithrombin III, 67.2%; fibrin (ogen) degradation products, 29.50 mg/L; D-dimer, 6.81 mg/L. Tumor markers: alpha-fetoprotein, >1204 ng/mL; carcinoembryonic antigen, 6.85 ng/mL; CA 125, 490 U/mL; ferritin, 1368 ng/mL; HBV DNA, <20 IU/mL. Upper abdominal MRI revealed an abnormally enhancing mass measuring 185 × 145 × 145 mm in the right hepatic lobe, accompanied by perihepatic fluid accumulation. The diagnosis was consistent with HCC. Liver function assessment yielded a Child-Pugh score of 8 points, corresponding to Grade B.

**Figure 1:**
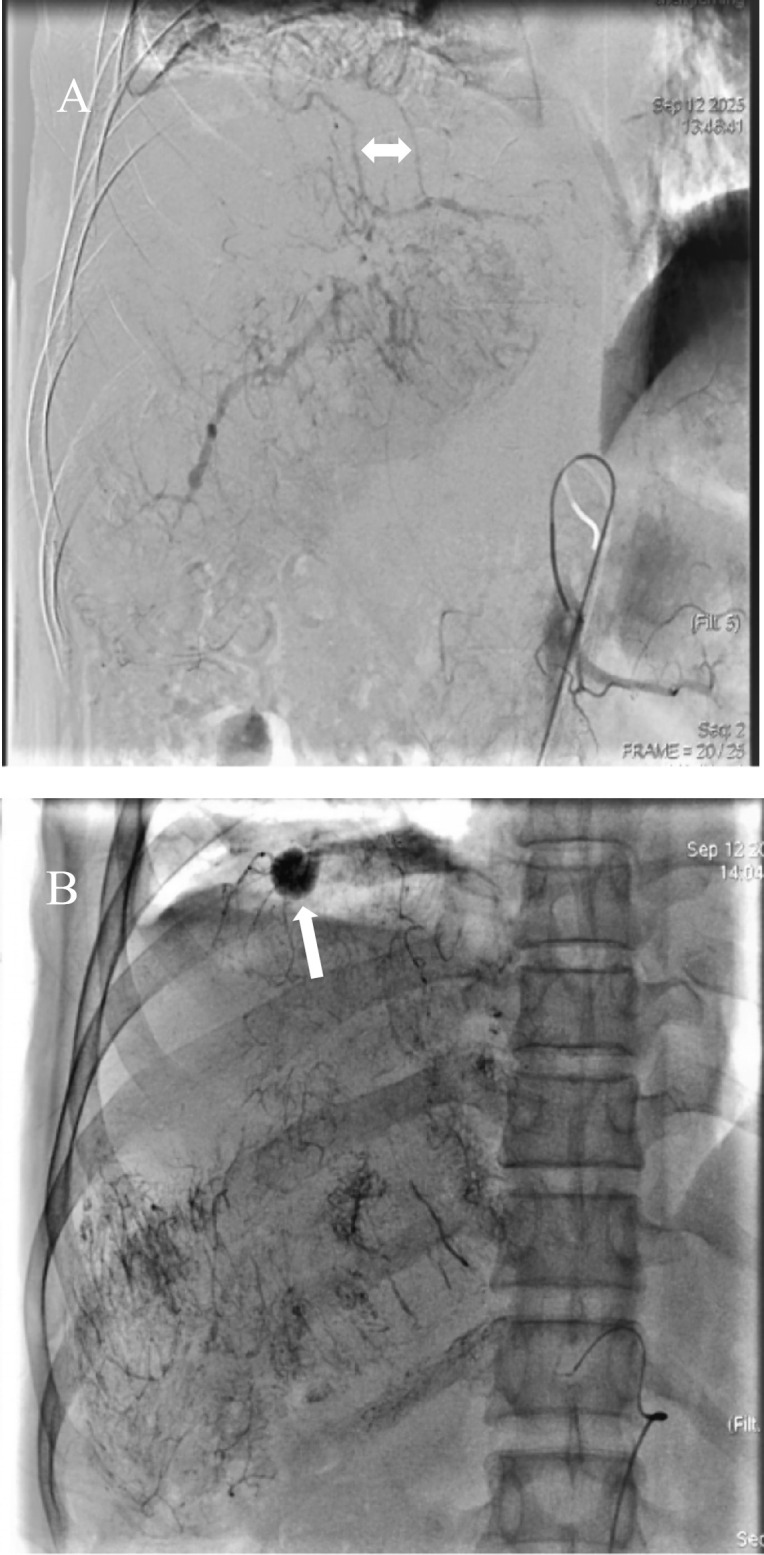
Angiography of hepatocellular carcinoma. **(A)** Intraoperative angiography revealed tumor supply from the right inferior phrenic artery (white arrow), with no evidence of an arteriovenous fistula. **(B)** Post-chemoembolization angiography demonstrated a vascular lake in the superior portion of the tumor (white arrow).

**Figure 2:**
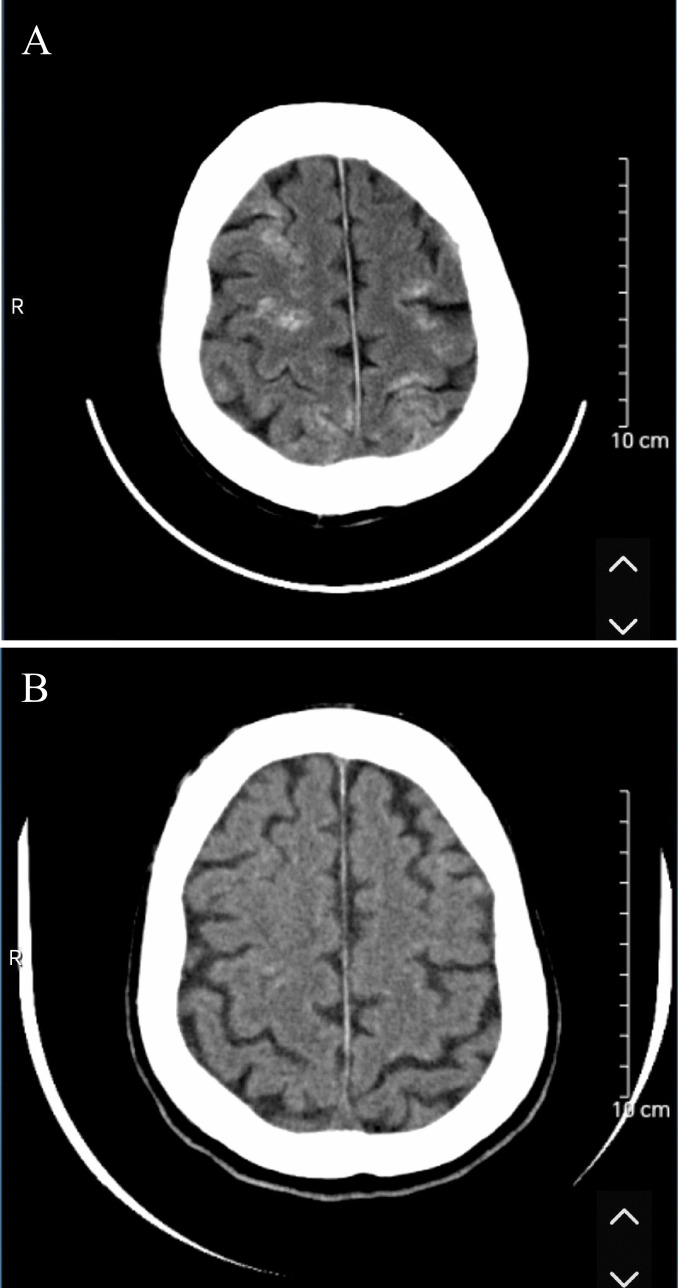
Head CT findings of cerebral lipiodol embolism. **(A)** One day after TACE: noncontrast cranial CT revealed multiple patchy hyperdense foci in the bilateral cerebral and cerebellar hemispheres. **(B)** One month after TACE: follow-up cranial CT demonstrated marked regression of the parenchymal hyperdense foci compared with the previous imaging.

TACE combined with targeted immunotherapy was administered to the patient. Angiography revealed a thickened and tortuous RHA with increased branching, along with prominent tumor staining indicative of primary HCC. A microcatheter (Terumo Corporation, Tokyo, Japan) was superselectively advanced into the tumor-feeding branch of the RHA. Intraoperative angiography identified the RIPA as the tumor’s blood supply source (**Figure 1A**), with no evidence of an arteriovenous fistula. During the procedure, a solution of oxaliplatin (150 mg diluted to 100 ml) plus raltitrexed (4 mg diluted to 100 mL) was first administered via perfusion. This was followed by embolization using an emulsion consisting of 15 mL of lipiodol (Lipiodol; Terumo Corporation, Tokyo, Japan) and pirarubicin, delivered at a rate of 1 mL/min. Subsequently, supplementary embolization was performed with 10 mL of embolic microspheres (300–500 μm, Embospheres) and 5 mL of gelatin sponge particles (Gelpart; Nippon Kayaku Co., Ltd., Tokyo, Japan). Embolization angiography revealed near-total disappearance of tumor staining with the emergence of vascular lakes (**Figure 1B**), without evidence of lipiodol migration. One day after TACE, the patient developed drowsiness, generalized fatigue, and inability to stand or walk. Physical examination revealed muscle strength of grade 4+ in both upper limbs, grade 3 in the left lower limb, and grade 3+ in the right lower limb. Physiological reflexes were intact, and pathological reflexes were negative. Head CT demonstrated multiple patchy hyperdense foci in the bilateral cerebral and cerebellar hemispheres. The ventricular system exhibited normal size, morphology, and architecture, with midline structures remaining centrally located. A diagnosis of CLE was established (**Figure 2A**). Chest CT revealed no evidence of lipiodol deposition, and there were no clinical manifestations indicative of pulmonary embolism.

The patient received anticoagulant therapy, agents for promoting blood circulation and removing blood stasis, and intravenous infusion of lipid emulsion. Following treatment, the patient’s level of consciousness and muscle strength gradually improved, leading to discharge on postoperative day 10. At the time of discharge, muscle strength was graded as 5 in both upper limbs, 5− in the left lower limb, and 5 in the right lower limb. The patient was able to ambulate independently and walk a distance of 100–200 meters. At one month after TACE, muscle strength had normalized to grade 5 in all four limbs, with restored ambulatory function. Cranial CT imaging showed a marked reduction in multiple hyperdense foci within the brain parenchyma compared with previous findings (**Figure 2B**).

## Results

A systematic literature search was conducted in the China National Knowledge Infrastructure (CNKI) and PubMed databases using the search terms “lipiodol cerebral embolism”, “cerebral lipiodol embolism”, “CLE”, “TACE-related cerebral embolism”, “liver cancer”, “hepatocellular carcinoma”, “HCC”, “transcatheter arterial chemoembolization”, and “TACE”, along with their MeSH or subject heading equivalents. After applying predefined eligibility criteria, 31 peer-reviewed publications were included, collectively reporting 49 confirmed cases of CLE following TACE (**Table 1**) (7–37).

**Table 1:** Clinical and radiological manifestation of 49 patients with CLE associated with TACE.

References	Case No.	Gender	Age	HCC description	Maximum diameter of HCC (cm)	Invade	Virus hepatitis	Feeding artery	Frequency of TACE	Dose of lipiodol (ml)	Time of CLE (m/h/d)	Neurological symptoms	Pulmonary involvement	Locus of lipiodol deposition in the brain	Treatment	Outcome	Causes of CLE
Yoo 2004 (7)	2	M	52	ND	ND	ND	ND	HA	2	35	Intraoperative	Yes	Yes	Widespread	SST	Survive	AV shunt
Yoo 2004 (7)	3	M	58	ND	ND	ND	ND	ND	ND	8	Intraoperative	Yes	Yes	Widespread	SST	Survive	AV shunt
Yoo 2004 (7)	4	M	56	ND	ND	ND	ND	ND	3	ND	Intraoperative	Yes	Yes	Widespread	SST	Survive	AV shunt
Anming 2001 (8)	1	M	51	Right liver	5	Diaphragm	ND	RHA+PA	2	ND	3h postoperatively	Yes	Yes	Widespread	SST	Survive	AV shunt
Ishimaru 2018 (9)	28	M	63	Left liver	18	ND	HBV	LHA+LIPA	2	16	Immediately	Yes	Yes	Widespread	SST	Survive	Vascular lake
Chao 2011 (10)	5	M	55	Superior part of the liver	ND	PV	ND	HA	3	30	2d postoperatively	Yes	ND	Widespread	SST	Survive	AV shunt
Chao 2011 (10)	6	F	55	Superior part of the huge liver	ND	ND	ND	RIPA	1	20	8h postoperatively	Yes	Yes	Widespread	SST	Die	IPA
Jianku 2011 (11)	7	F	57	Recurrent right liver	10	ND	HBV	PHA	5	15	3h postoperatively	Yes	ND	Widespread	SST	Survive	AV shunt
Jianku 2011 (11)	8	M	66	Right lobe	ND	ND	ND	RHA	1	12	6h postoperatively	Yes	Yes	Widespread	SST	Survive	AV shunt
Yongchuang 2010 (12)	9	F	71	Huge right lobe	ND	ND	ND	IA+RHA	3	20	Intraoperative	Yes	Yes	Widespread	SST	Die	Intercostal artery
Yongchuang 2010 (12)	10	M	62	Huge	ND	ND	ND	PHA	3	70	Immediately	Yes	Yes	Widespread	SST	Survive	AV fistula
Yongchuang 2010 (12)	11	M	69	Huge	ND	ND	HBV	PHA	2	20	Immediately	Yes	Yes	Widespread	SST	Survive	AV fistula
Shixiang 2010 (13)	12	F	68	Left lobe、adjacent to the diaphragm	15	ND	ND	LHA	2	25	Immediately	Yes	ND	Widespread	Fat emulsion +SST	Die	AV shunt
Haitao 2012 (14)	13	M	64	Right liver	ND	Diaphragm	HBV	RHA+GDA	2	15	31h postoperatively	Yes	Yes	Widespread	SST	Survive	AV shunt
Haitao 2012 (14)	14	M	52	Huge right lobe	ND	Diaphragm	ND	RHA	2	40	2h postoperatively	Yes	Yes	Widespread	SST	Survive	AV shunt
Han 2005 (15)	15	M	47	Right liver	10.3	ND	ND	PHA+ RIPA	4	10	5h postoperatively	Yes	Yes	Widespread	SST	Survive	IPA
Gongjie 2005 (16)	16	F	65	Right liver, adjacent to the diaphragm	12	ND	ND	PHA	3	35	6h postoperatively	Yes	Yes	Widespread	Fat emulsion +SST	Die	AV shunt
Gongjie 2005 (16)	17	M	57	Superior part of the right liver、adjacent to the diaphragm	7	ND	HBV	RHA+PA	11	10	10h postoperatively	Yes	ND	Widespread	Fat emulsion +SST	Survive	AV fistula+ IPA
Gongjie 2005 (16)	18	M	63	Right liver、adjacent to the diaphragm	18	Lung	ND	RHA	3	40	6h postoperatively	Yes	Yes	Widespread	SST	Die	AV fistula
Peng 2010 (17)	19	M	ND	Right liver	11	ND	HBV	RHA	3	15	Nocturnal Postoperative	Yes	Yes	Widespread	SST	Die	AV fistula
Xiaobao 2004 (18)	20	M	ND	Huge right liver、adjacent to the diaphragm	ND	ND	HBV	RHA+IPA	2	ND	Intraoperative	Yes	ND	Widespread	SST	Die	IPA
Changsheng 2016 (19)	21	F	65	Huge right liver、adjacent to the diaphragm	12	ND	ND	PHA	3	35	6h postoperatively	Yes	Yes	Widespread	Fat emulsion +SST	Die	AV shunt+ PFO+AV fistula
Changsheng 2016 (19)	22	M	57	Right liver	7	ND	HBV	RHA+PA	11	10	10h postoperatively	Yes	ND	Widespread	Fat emulsion +SST	Survive	AV shunt+ PFO+AV fistula
Piljin 2009 (20)	23	M	67	Single	3	ND	HBV	RIPA	4	5	Immediately	Yes	Yes	Widespread	SST	Survive	RIPA +PFO resulting in right-to-left shunt.
Kim 2009 (21)	24	M	67	Left liver、 Multiple	4.3	ND	ND	HA+RIPA+PHA	4	5	Immediately	Yes	NO	Widespread	SST	Survive	AV shunt+Intracardiac right-to-left shunt
Kim 2009 (21)	25	F	63	Huge right liver	ND	ND	ND	RHA+ RIPA	3	10	Immediately	Yes	ND	Widespread	SST	Survive	Intrapulmonary/Intracardiac Right-to-Left Shunt
Zhi 2011 (22)	26	M	62	Right liver	9.7	ND	HBV	RHA	3	80	Intraoperative	Yes	ND	Widespread	SST	Survive	AV shunt
Zhi 2011 (22)	27	F	71	Right liver	ND	ND	ND	RHA+IPA	3	50	Intraoperative	Yes	ND	Widespread	SST	Die	AV shunt
Zhongzhi 2012 (23)	29	M	54	Right liver	13	Diaphragm	HBV	RHA+RIPA	1	30	20min postoperatively	Yes	ND	Widespread	SST	Survive	AV shunt
Quanmin 2015 (24)	30	M	71	Right liver	10.1	ND	HBV	RAA	5	20	Intraoperative	Yes	ND	Widespread	SST	Survive	Abdominal trauma
Lu 2010 (25)	31	M	41	ND	ND	PV	ND	PHA	2	30	Intraoperative	Yes	Yes	Widespread	SST	Survive	AV fistula
Bánsághi 2013 (26)	32	M	52	Right liver	18	Diaphragm	ND	RIPA+RHA	1	50	30min postoperatively	Yes	Yes	Widespread	SST	Die	AV shunt
Chihchen 2015 (27)	33	F	66	Recurrent HCC	ND	ND	HBV	RHA+LIPA	5	20	Immediately	Yes	Yes	Widespread	SST	Survive	AV shunt
Zach 2012 (28)	34	M	66	Multiple	11.5	ND	ND	RHA	1	4	85min postoperatively	Yes	Yes	Widespread	SST	Die	AV shunt
Karapanayiotides 2009 (29)	35	M	71	ND	ND	ND	ND	ND	4	ND	Intraoperative	Yes	ND	Widespread	SST	Survive	AV shunt
Jianjun 2009 (30)	36	M	36	Huge right liver	ND	Diaphragm	HBV	RHA	2	40	2h postoperatively	Yes	Yes	Widespread	SST	Survive	AV shunt
Jianjun 2009 (31)	37	F	51	Huge right liver	ND	ND	HBV	RHA	2	40	69h postoperatively	Yes	Yes	Widespread	SST	Survive	AV shunt
Haijui 2015 (32)	38	M	51	Multiple	13	ND	HBV	RHA+LHA	4	30	≤6h postoperatively	Yes	Yes	Widespread	SST	Survive	AV shunt
Haijui 2015 (32)	39	F	73	Multiple	19	ND	HCV	RIPA	4	15	≤6h postoperatively	Yes	Yes	Widespread	SST	Survive	AV shunt
Haijui 2015 (32)	40	F	67	Multiple	6	ND	HCV	LGA	11	10	≤6h postoperatively	Yes	Yes	Widespread	SST	Survive	AV shunt
Haijui 2015 (32)	41	F	54	Multiple	3	ND	HBV	LIPA	4	90	≤6h postoperatively	Yes	Yes	Widespread	SST	Die	AV shunt
Haijui 2015 (32)	42	M	63	Single	14	ND	HBV	RSGA	3	50	≤6h postoperatively	Yes	Yes	Widespread	SST	Survive	AV shunt
Haijui 2015 (32)	43	M	52	Multiple	17	ND	HBV	RHA	2	30	≤6h postoperatively	Yes	Yes	Widespread	SST	Survive	AV shunt
Haijui 2015 (32)	44	M	72	Multiple	10	ND	HBV	RHA	2	20	≤6h postoperatively	Yes	Yes	Widespread	SST	Die	AV shunt
Choi 2008 (33)	45	F	62	Right liver	15	ND	HBV	RIPA+RHA	3	30	Immediately	Yes	Yes	Widespread	SST	Survive	IPA
Lee 2010 (34)	46	M	44	Superior surface of the right liver	ND	Diaphragm	ND	PHA+LBA	4	ND	Immediately	Yes	Yes	Widespread	SST	Survive	Bronchial Artery
Renghong 2005 (35)	47	F	81	Right liver	14	Diaphragm	ND	RIPA+RIMA	2	20	Immediately	Yes	Yes	Widespread	SST	Die	Right-to-Left Shunt
Ishikawa 2013 (36)	48	M	62	Right liver、adjacent to the diaphragm	16	PV	ND	ND	3	ND	1d postoperatively	Yes	ND	Widespread	SST	Survive	AV shunt
Matsumoto 2007 (37)	49	F	70	Recurrent huge HCC	ND	Diaphragm	ND	RHA+MHA+ IPA+RCA	1	12	Immediately	Yes	NO	Widespread	SST	Die	AV shunt+Right-to-Left Shunt

F, female; M, male; ND, not described; huge tumor diameter ≥10 cm; multiple = ≥3 lesions; single = 1–2 lesions; CLE, cerebral lipiodol embolism; HCC, hepatocellular carcinoma; TACE, transcatheter arterial chemoembolization; HA, hepatic artery; PA, phrenic artery; RHA, right hepatic artery; IA, intercostal artery; LHA, left hepatic artery; MHA, middle hepatic artery; RCA, right renal capsular artery; IPA, inferior phrenic artery; RIPA, right inferior phrenic artery; RAA, right adrenal artery; LGA, left gastric artery; LIPA, left inferior phrenic artery; RSGA, right superior gluteal artery; GDA, gastroduodenal artery; PHA, proper hepatic artery; PV, portal vein; LBA, left bronchial artery; RIMA, right internal mammary artery; SGA, superior gluteal artery; PFO, patent foramen ovale; AV shunt, arteriovenous shunt; AV fistula, arteriovenous fistula; SST, symptomatic supportive therapy.

Among the 49 patients, 45 (91.8%) developed symptoms suggestive of acute cerebral ischemia within 12 hours after TACE, with nonspecific clinical presentations including headache, limb weakness, hypoesthesia, aphasia, urinary incontinence, altered consciousness, visual disturbances, limb paralysis, or even fatal outcomes. Neuroimaging revealed extensive lipiodol deposition in the cerebral cortex, basal ganglia, thalamus, gray–white matter junction, lateral ventricles, brainstem, and small extracranial scalp vessels in some patients. Concurrent pulmonary embolism was observed in 34 cases (69.4%), predominantly manifesting as chest tightness, cough, dyspnea, and hypoxic respiratory failure. Clinical management of CLE primarily involves symptomatic and supportive care, including neuroprotective interventions, cerebral perfusion optimization, and respiratory support. Five patients received intravenous lipid emulsion therapy. Of the 49 patients, 15 (30.6%) ultimately died of multiple organ failure. Most surviving patients achieved complete neurological recovery with symptomatic and supportive treatment, without residual neurological sequelae.

Among the 49 patients, 6 developed CLE after the first TACE procedure, and 13 developed CLE after ≥3 TACE procedures. Lipiodol dosage was ≥20 mL in 27 cases; tumor diameter was ≥10 cm in 30 cases, including 8 cases (16.3%) of multinodular HCC; 21 patients had HBV infection and 2 had chronic HCV infection; tumor invasion of the diaphragm was observed in 9 cases, and portal vein invasion in 3 cases; the inferior phrenic artery contributed to tumor blood supply in 16 cases.

Five cases had a clear etiology: patent foramen ovale (PFO) in one case (2.0%), intracardiac right-to-left shunt in two cases (4.1%), pulmonary artery–portal vein fistula in one case (2.0%), intercostal artery–pulmonary vein fistula in one case (2.0%), and bronchial artery–pulmonary vein fistula in one case (2.0%). Additionally, an intratumoral “vascular lake” sign was observed in one case (2.0%), with lipiodol draining via the pericardiacophrenic vein into the systemic venous circulation.

## Discussion

Liver cancer is one of the most common malignancies in China. According to data released by the National Cancer Center of China in 2022, the incidence rate of PLC ranked fourth among malignant tumors nationwide, and it ranked second in cancer-related deaths (38). PLC is characterized by an insidious onset, and the majority of patients with this disease are diagnosed at the middle and advanced stages (5).

TACE is the most widely adopted transarterial interventional approach for treating HCC (39, 40), which involves embolizing tumor-feeding arteries with lipiodol emulsion, optionally combined with particulate embolic agents (e.g., gelatin sponge particles, blank microspheres, polyvinyl alcohol granules) or drug-eluting microspheres (41). CLE refers to a pathological condition in which lipiodol—commonly employed as an embolic agent, contrast medium, or drug carrier during interventional diagnostic or therapeutic procedures—inadvertently enters the cerebral vasculature due to various factors. This results in mechanical occlusion of cerebral blood vessels, potentially accompanied by secondary endothelial inflammation, platelet aggregation, and thrombus formation. The process culminates in ischemic and hypoxic damage to cerebral tissue. Kusumomo et al. first reported CLE following lymphography with lipiodol in 1991 (42), and Li et al. published the first reported case of CLE associated with TACE in China in 2001 (8).

Mechanistic speculation for this case: This case describes a patient with intermediate-stage huge HCC who underwent initial TACE, during which a 15-mL lipiodol-chemotherapeutic emulsion was administered. The tumor is primarily supplied by the RHA, with collateral perfusion from the RIPA. Angiographic observation during TACE revealed vascular lakes—characterized by localized accumulation of the lipiodol-chemotherapeutic mixture, manifesting as abnormal, blood-filled cavities resembling “extravasation” within the tumor. Some vascular lakes exhibit identifiable drainage veins (9), though their efferent pathways remain inadequately characterized in most cases. The absence of pulmonary lipiodol deposition in this patient may be attributed to the formation of an arteriovenous shunt via neovascular communications between the vascular lake and adjacent veins. Alternatively, minute hepatic arteriovenous fistulas within the massive tumor could permit lipiodol entry into the systemic circulation.

The pathogenic mechanism underlying CLE remains incompletely elucidated to date. A review of relevant literature suggests that the entry of lipiodol into cerebral vessels may be attributed to aberrant vascular communications, such as intracardiac right-to-left shunts or intrapulmonary arteriovenous shunts; or to repeated TACE sessions or excessively rapid or overdosed administration of lipiodol. 1. Abnormal Vascular Communication: 1) Intracardiac right-to-left shunts, such as PFO and ventricular septal defect. Research findings have established an association between PFO and left-sided circulatory thromboembolism (43). Moreover, PFO is observed in up to 40% of stroke patients with undetermined etiology (44). Patients with HCC frequently present with concomitant hepatic arteriovenous fistulas, through which lipiodol may reflux into the right atrium. Owing to intracardiac right-to-left shunting, lipiodol can bypass the buffering and filtering effects of the pulmonary circulation, directly entering the systemic circulation and causing non-target lipiodol embolization. However, in previously reported cases of CLE, the pathway from the pulmonary artery to the left atrium remains unverified (9). 2) Intrapulmonary arteriovenous shunting. An anomalous connection forms between the tumor-feeding artery and pulmonary veins, allowing lipiodol from the tumor vasculature to enter the systemic circulation directly. The inferior phrenic artery serves as the most crucial extrahepatic blood supply vessel. In addition to its anastomoses with the proper hepatic artery, it also forms connections with intrapulmonary arteries and veins, bronchial arteries, pericardiac arteries, and internal thoracic arteries (5). When tumors are located adjacent to the diaphragm or pulmonary tissue, microscopic shunts are prone to develop between the hepatic arteries and pulmonary veins within the tumor. This can lead to cerebral embolism when lipiodol enters the left ventricle (28). 2. TACE: 1) Multiple TACE procedures may induce neovascularization and promote the formation of aberrant vascular pathways, thereby increasing the risk of CLE occurrence (10). 2) Excessive administration rate or dosage of lipiodol may elevate intravascular pressure, thereby facilitating the passage of lipiodol or its admixtures through pulmonary capillaries into the systemic circulation (45). Furthermore, the study by Kishi et al. (46) demonstrated a significant positive correlation between the administered lipiodol dosage and the amount reaching the systemic circulation.

After hepatic artery injection of lipiodol, 74.38%–91.36% remains in the hepatic parenchyma and tumor microvascular bed; 2.45%–8.57% enters the pulmonary circulation via hepatic arteriovenous shunts and is temporarily stored; and a small fraction distributes to organs of the mononuclear phagocyte system (MPS), such as the spleen and bone marrow. The retention time of lipiodol in tumor tissue is significantly longer than that in normal liver tissue (47, 48). Its effective half-life is 3.5–4.35 days in normal liver tissue, approximately 5.5 days in tumor tissue, and 4.0–5.23 days in lung tissue; its plasma clearance half-life ranges from 2 to 3 days (47–49). Iodine is primarily excreted by the kidneys in an inorganic form: approximately 30%–50% is eliminated in urine within 7 days post-administration; fecal excretion is minimal (≈3% within 5 days); biliary excretion and lymphatic clearance constitute minor pathways, with no significant enterohepatic circulation observed (47–49). A small portion of lipiodol may persist long-term in the lungs, liver, adipose tissue, and MPS organs, where it is slowly metabolized and cleared by macrophages. This storage—which can last from months to years—represents an important pathological basis for the long-term complications associated with lipiodol-induced ectopic embolization (47–49). Primary Liver Cancer Diagnosis and Treatment Guidelines (2024 Edition) (41) recommend that lipiodol and chemotherapeutic agents be thoroughly emulsified prior to administration. The typical single dosage ranges from 5 to 20 mL, with a maximum limit not exceeding 30 mL. For drug-eluting bead transarterial chemoembolization (DEB-TACE), microspheres with particle sizes of 100–300 µm or 300–500 µm are commonly employed. To minimize the risk of non-target embolization, the recommended injection rate for drug-eluting microspheres is 1 mL/min.

In addition to the aforementioned mechanisms, a large tumor is one of the high-risk factors for CLE. The large tumor exhibits rich intratumoral vascularity, with potential invasion into adjacent organs and blood vessels leading to anomalous vascular communications. During TACE, individualized dosing of lipiodol is required; a relatively high dose is often necessary to achieve complete embolization of the tumor vasculature. Should the initial TACE procedure yield suboptimal outcomes, multiple sessions of TACE may be warranted for adequate therapeutic efficacy.

In summary, although most causes of CLE are inherently unavoidable, clinicians should closely monitor abnormal vascular shunting—particularly arteriovenous or arterioportal communications—in high-risk patients undergoing TACE, and tailor therapeutic strategies accordingly to mitigate the risk of CLE. The clinical features and imaging manifestations summarized in this study facilitate timely diagnosis and appropriate management of CLE.

As a case report with literature review, this study has unavoidable limitations, including a small number of cases and potential heterogeneity among included studies. In addition, adequate comparisons with studies conducted in other countries were not performed due to differences in population characteristics and clinical practice patterns. Thus, the conclusions should be treated prudently, and more high−quality, multi−center, cross−national research is warranted to validate the present findings.

## Data Availability

The original contributions presented in the study are included in the article/supplementary material. Further inquiries can be directed to the corresponding author.

## References

[1] Zhou M Wang H Zeng X Yin P Zhu J Chen W . Mortality, morbidity, and risk factors in China and its provinces, 1990-2017: a systematic analysis for the Global Burden of Disease Study 2017. Lancet. (2019) 394:1145–58. doi: 10.1016/S0140-6736(19)30427-1. PMID: 31248666 PMC6891889

[2] Bray F Ferlay J Soerjomataram I Siegel RL Torre LA Jemal A . Global cancer statistics 2018: GLOBOCAN estimates of incidence and mortality worldwide for 36 cancers in 185 countries. CA Cancer J Clin. (2018) 68:394–424. doi: 10.3322/caac.21492. PMID: 30207593

[3] Cao GW . Epidemiological characteristics and precise prophylaxis and control of primary liver cancer in China. J Guangxi Med Univ. (2024) 41:1455–63. doi: 10.16190/j.cnki.45-1211/r.2024.11.002

[4] Xian LF Fang LT Liu WB Zhao P Chen YF Cao GW . The prevalence status, main pathogenic mechanisms and prevention strategies of primary liver cancer. Chin J Oncol Prev Treat. (2022) 14:320–8. doi: 10.3969/j.issn.1674-5671.2022.03.13

[5] Chen MXJ . Pulmonary and cerebral lipiodol embolism after transcatheter arterial chemoembolization of hepatocellular carcinoma. (Doctoral dissertation) J Hebei Med Univ. (2018). doi: 10.1111/j.1365-2354.2005.00609.x. PMID: 16274465

[6] Chen SB Yu XX . Risk factors of pulmonary lipiodol embolism after TACE for hepatocellular carcinoma. J Hepatopancreatobil Surg. (2017) 29:276–80. doi: 10.1212/01.wnl.0000132645.23611.2d, PMID: 15249637

[7] Yoo KM Yoo BG Kim KS Lee SU Han BH . Cerebral lipiodol embolism during transcatheter arterial chemoembolization. Neurology. (2004) 63:181–3. doi: 10.1212/01.wnl.0000132645.23611.2d. PMID: 15249637

[8] Li AM Wang RP Zhou S Wang XJ . Cerebral lipiodol embolism caused by hepatic chemoembolization: one case report. Oncoradiology. (2001) 10:3. Available online at: https://lib.cqvip.com/Qikan/Article/Detail?id=5545536.

[9] Ishimaru H Morikawa M Sakugawa T Sakamoto I Motoyoshi Y Ikebe Y . Cerebral lipiodol embolism related to a vascular lake during chemoembolization in hepatocellular carcinoma: A case report and review of the literature. World J Gastroenterol. (2018) 24:4291–6. doi: 10.3748/wjg.v24.i37.4291. PMID: 30310262 PMC6175758

[10] Liu C Guan S Li MX Ma N Zhang JH Hu XB . Cerebral lipiodol embolism caused by interventional therapy for hepatic tumor: report of two cases with literature review. J Intervent Radiol. (2011) 20:135–7. doi: 10.1111/liv.12511. PMID: 24571486

[11] Du JK He WH Zhang MD Li GH . Cerebral Lipiodol Embolism caused by hepatic cancer interventional embolization: Two case reports. Clin J Med Offic. (2011) 39:814–5. Available online at: https://lib.cqvip.com/Qikan/Article/Detail?id=38983283.

[12] Chang YC Ni CF Liu YZ Zou JW Chen L . Pulmonary and cerebral lipiodol embolism during transcatheter arterial chemoembol ization in the treatment of hepatocellular carcinoma. Chin J Interv Imaging Ther. (2010) 7:221–4. doi: 10.13929/j.1672-8475.2010.03.031

[13] Li SX Xia GW Yu T . TACE complicated with cerebral lipiodol embolism in a liver cancer patient. Contemp Med. (2010) 16:241–2. doi: 10.3969/j.issn.1009-4393.2010.11.045

[14] He HT Liu S Liu JQ Zhou GY . Cerebral Lipiodol embolism occurred after transcatheter arterial chemoembolization for hepatocellular carcinoma: report of two cases with literature review. J Intervent Radiol. (2012) 21:682–4. Available online at: https://lib.cqvip.com/Qikan/Article/Detail?id=43024737.

[15] Wang H Wang JB Zhang GX Wang LC . Cerebral infarction after transarterial chemoembolization for hepatocellular carcinoma: a case report. J Intervent Radiol. (2005) 14 (04):442–3. doi: 10.3748/wjg.14.6425. PMID: 19009665 PMC2766131

[16] Li GJ Ren XG Sheng FG Xing XD Liu YY . Extensive cerebral terminal artery lipiodol embolism caused by primary hepatocellular carcinoma hepatic artery embolization: one case report. Clin J Radiol. (2005) 39 (09):1007–8. Available online at: https://lib.cqvip.com/Qikan/Article/Detail?id=20247541.

[17] Du P Liu JY Peng WF Lin HJ Ma Y Fan W . Cerebral lipiodol embolism: a case report and review of the literature. Neural Injury Funct Reconstruction. (2010) 5:381–3. doi: 10.3870/sjsscj.2010.05.015

[18] Li XB Xu GB Wang F Zhou S Wang XJ . Cerebral infarction caused by hepatic cancer interventional embolization: one case report. Clin J Radiol. (2004) 38 (8):894–5. Available online at: https://lib.cqvip.com/Qikan/Article/Detail?id=10633663.

[19] Shi CS Yang Q Shi ZJ Zheng BR Yu XX . Cerebral lipiodol embolism after transarterial chemoembolization for hepatic carcinoma: two case reports. Chin J Inter Rad (Electronic Edition). (2016) 4:243–5. doi: 10.3877/cma.j.issn.2095-5782.2016.04.014

[20] Chung PJ Park SY Kim YI Yoon KW Cho SB Choi SK . Cerebral lipiodol embolism after transcatheter arterial chemoembolization of hepatocellular carcinoma. Korean J Gastroenterol. (2009) 54:130–4. doi: 10.4166/kjg.2009.54.2.130. PMID: 19696542

[21] Kim JT Heo SH Choi SM Lee SH Park MS Kim BC . Cerebral embolism of iodized oil (lipiodol) after transcatheter arterial chemoembolization for hepatocellular carcinoma. J Neuroimaging. (2009) 19:394–7. doi: 10.1111/j.1552-6569.2009.00380.x. PMID: 19496902

[22] Li Z Ni RF Busireddy KK Jin YH Zhao X Li MM . Cerebral lipiodol embolism following transcatheter arterial chemoembolization for hepatocellular carcinoma: a report of two cases and literature review. Chin Med J (Engl). (2011) 124:4355–8. doi: 10.3760/cma.j.issn.0366-6999.2011.24.014, PMID: 22340413

[23] Jia ZZ Tian F Jiang GM . Cerebral lipiodol embolism after transarterial chemoembolization for hepatic carcinoma: a case report. World J Gastroenterol. (2012) 18:4069–70. doi: 10.3748/wjg.v18.i30.4069. PMID: 22912560 PMC3421436

[24] Nie Q Wu H Guo P Ge J Qiu Y . Cerebral lipiodol embolism following abdomen trauma in a patient with hepatocellular carcinoma treated with transcatheter arterial chemoembolization. Acta Neurol Belg. (2015) 115:459–61. doi: 10.1007/s13760-014-0376-x. PMID: 25319130

[25] Wu L Yang YF Liang J Shen SQ Ge NJ Wu MC . Cerebral lipiodol embolism following transcatheter arterial chemoembolization for hepatocellular carcinoma. World J Gastroenterol. (2010) 16:398–402. doi: 10.3748/wjg.v16.i3.398. PMID: 20082490 PMC2807965

[26] Bánsághi Z Kaposi PN Lovas G Szentmártoni G Várallyay G Bata P . Cerebral iodized lipid embolization via a pulmonary arteriovenous shunt: rare complication of transcatheter arterial embolization for hepatocellular carcinoma. World J Surg Oncol. (2013) 11:122. doi: 10.1186/1477-7819-11-122. PMID: 23721061 PMC3681566

[27] Wan CC Liu KL . Cerebral lipiodol embolism. Liver Int. (2015) 35:673. doi: 10.1111/liv.12511. PMID: 24571486

[28] Zach V Rapaport B Yoo JY Goldfeder L Weinberger J . Multiple ischemic strokes after transcatheter arterial chemoembolization for hepatocellular carcinoma with a radiographic and pathological correlate. J Stroke Cerebrovasc Dis. (2012) 21:217–24. doi: 10.1016/j.jstrokecerebrovasdis.2010.08.001. PMID: 21036627

[29] Karapanayiotides T Goulis J Theodorou A Anastasiou A Georgiadis G Ilonidis G . Lipiodol brain embolism during hepatic transcatheter arterial chemoembolization. J Neurol. (2009) 256:1171–3. doi: 10.1007/s00415-009-5066-x. PMID: 19252772

[30] Wu JJ Chao M Zhang GQ Li B Dong F . Pulmonary and cerebral lipiodol embolism after transcatheter arterial chemoembolization (corrected) in hepatocellular carcinoma. World J Gastroenterol. (2009) 15:633–5. doi: 10.3748/wjg.15.633. PMID: 19195069 PMC2653354

[31] Wu JJ Chao M Zhang GQ Li B . Delayed cerebral lipiodol embolism after transcatheter arterial chemoembolization of hepatocellular carcinoma. Chin Med J (Engl). (2009) 122:878–80. doi: 10.3748/wjg.v16.i3.398. PMID: 19493407

[32] Chu HJ Lee CW Yeh SJ Tsai LK Tang SC Jeng JS . Cerebral lipiodol embolism in hepatocellular carcinoma patients treated with transarterial embolization/chemoembolization. PloS One. (2015) 10:e0129367. doi: 10.1371/journal.pone.0129367. PMID: 26107693 PMC4481105

[33] Choi CS Kim KH Seo GS Cho EY Oh HJ Choi SC . Cerebral and pulmonary embolisms after transcatheter arterial chemoembolization for hepatocellular carcinoma. World J Gastroenterol. (2008) 14:4834–7. doi: 10.3748/wjg.14.4834. PMID: 18720550 PMC2739351

[34] Lee CS Kim SJ Choi JW Choi CG Lee DH . Cerebral lipiodol embolism proven by dual-energy computed tomography: a case report. J Comput Assist Tomogr. (2010) 34:105–6. doi: 10.1097/RCT.0b013e3181b382f8. PMID: 20118731

[35] Wu RH Tzeng WS Chang CM . Iodized oil embolization to brain following transcatheter arterial embolization of liver. J Gastroenterol Hepatol. (2005) 20:1465–7. doi: 10.1111/j.1440-1746.2005.03412.x. PMID: 16105143

[36] Ishikawa T Kubota T Abe H Toduka Y Horigome R Watanabe Y . Case of cerebral lipiodol embolism after repeated transcatheter arterial chemoembolization of hepatocellular carcinoma. Hepatol Res. (2013) 43:1251–2. doi: 10.1111/hepr.12074. PMID: 24580681

[37] Matsumoto K Nojiri J Takase Y Egashira Y Azama S Kato A . Cerebral lipiodol embolism: a complication of transcatheter arterial chemoembolization for hepatocellular carcinoma. Cardiovasc Intervent Radiol. (2007) 30:512–4. doi: 10.1007/s00270-006-0092-x. PMID: 17171304

[38] Han B Zheng R Zeng H Wang S Sun K Chen R . Cancer incidence and mortality in China, 2022. J Natl Cancer Center. (2024) 4:47–53. doi: 10.1016/j.jncc.2024.01.006. PMID: 39036382 PMC11256708

[39] Lencioni R de Baere T Soulen MC Rilling WS Geschwind JF . Lipiodol transarterial chemoembolization for hepatocellular carcinoma: A systematic review of efficacy and safety data. Hepatology. (2016) 64:106–16. doi: 10.1002/hep.28453. PMID: 26765068

[40] Llovet JM Bruix J . Systematic review of randomized trials for unresectable hepatocellular carcinoma: Chemoembolization improves survival. Hepatology. (2003) 37:429–42. doi: 10.1053/jhep.2003.50047. PMID: 12540794

[41] National Health Commission of the People’s Republic of China . Guidelines for the Diagnosis and Treatment of Primary Liver Cancer (2026 Edition). Chin J Dig Surg. (2026) 25(4):423–82. doi: 10.3760/cma.j.cn115610-20260409-00187, PMID: 30704229

[42] Kusumoto S Imamura A Watanabe K . Case report: the incidental lipid embolization to the brain and kidney after lymphography in a patient with Malignant lymphoma: CT findings. Clin Radiol. (1991) 44:279–80. doi: 10.1016/s0009-9260(05)80199-0. PMID: 1959309

[43] Cramer SC Rordorf G Maki JH Kramer LA Grotta JC Burgin WS . Increased pelvic vein thrombi in cryptogenic stroke: results of the Paradoxical Emboli from Large Veins in Ischemic Stroke (PELVIS) study. Stroke. (2004) 35:46–50. doi: 10.1161/01.str.0000106137.42649.ab. PMID: 14657451

[44] Lechat P Mas JL Lascault G Loron P Theard M Klimczac M . Prevalence of patent foramen ovale in patients with stroke. N Engl J Med. (1988) 318:1148–52. doi: 10.1056/nejm198805053181802. PMID: 3362165

[45] Yoon W Kim JK Kim YH Chung TW Kang HK . Bronchial and non-bronchial systemic artery embolization for life-threatening hemoptysis: a comprehensive review. Radiographics. (2002) 22:1395–409. doi: 10.1148/rg.226015180. PMID: 12432111

[46] Kishi K Sonomura T Satoh M Nishida N Terada M Shioyama Y . Acute toxicity of lipiodol infusion into the hepatic arteries of dogs. Invest Radiol. (1994) 29:882–9. doi: 10.1097/00004424-199410000-00004. PMID: 7852039

[47] Nakajo M Kobayashi H Shimabukuro K Shirono K Sakata H Taguchi M . Biodistribution and *in vivo* kinetics of iodine-131 lipiodol infused via the hepatic artery of patients with hepatic cancer. J Nucl Med. (1988) 29:1066–77. doi: 10.2967/jnumed.29.6.3206682 2836573

[48] Raoul JL Bourguet P Bretagne JF Duvauferrier R Coornaert S Darnault P . Hepatic artery injection of I-131-labeled lipiodol. Part I. Biodistribution study results in patients with hepatocellular carcinoma and liver metastases. Radiology. (1988) 168:541–5. doi: 10.1148/radiology.168.2.2839866. PMID: 2839866

[49] Chen GC Chen YX Xu PS Yang HZ Chen Y Lu QJ . A safety study on hepatic artery injection of lipiodol in beagle dogs. Chin J Clin Pharmacol Ther. (1988) 29 (06):648–51. Available online at: https://manu41.magtech.com.cn/Jweb_clyl/CN/abstract/abstract12738.shtml.

